# Association between self-reported gender-based discrimination and maternal mortality rates: results of an ecological multi-level analysis across nine countries in Sub-Saharan Africa

**DOI:** 10.1186/s12889-025-24861-z

**Published:** 2025-10-21

**Authors:** Clara Orduhan, Ruth Waitzberg, Manuela De Allegri, Bona Chitah, Jean-Paul Dossou, Charlestine Bob Elwange, Adama Faye, Sharon Fonn, Christabel Kambala, Shafiu Mohammed, Hamidou Niangaly, Chenjerai Sisimayi, Wilm Quentin

**Affiliations:** 1 Department of Health Care Management, Technische Universität Berlin, Straße des 17. Juni 135, 10623 Berlin, Germany; 2 Faculty of Medicine and University Hospital, Heidelberg Institute of Global Health, Heidelberg University, INF 130.3, 69120 Heidelberg, Germany; 3 Department of Economics, University of Zambia, Great East Road, Lusaka, Zambia; 4 Centre de Recherche en Reproduction Humaine et en Démographie, CNHU/HKM, Avenue Jean-Paul II, Cotonou, Benin; 5 Department of Sociology, Anthropology and Population Studies, Faculty of Social Sciences, Kyambogo University, Kampala, Uganda; 6 Institut de Santé et Développement, Université Cheikh Anta Diop de Dakar, Dakar, Sénégal; 7 School of Public Health, University of the Witwatersrand, Johannesburg, South Africa; 8 School of Public Health and Community Medicine, University of Gothenburg, Gothenburg, Sweden; 9 Environmental Health Department, University of Business and Applied Sciences, Private Bag 303, Blantyre 3, Chichiri, Blantyre, Malawi; 10 Health Systems and Policy Research Unit, Ahmadu Bello University, Zaria, Nigeria; 11 Institut National de Santé Publique, Hippodrome, Road of Koulikoro, Street 235, Gate 52, Bamako, 1771 Mali; 12 MSc Clinical Epidemiology, World Bank, Block 3, Arundel Office Park, 107 Norfolk Road, Mt. Pleasant, Harare, Harare, +263772810716 Zimbabwe; 13 Prof. of Planetary & Public Health, University of Bayreuth, Universitätsstraße 30, 95447 Bayreuth, Germany; 14 European Observatory on Health Systems and Policies, Department of Health Care Management, Technische Universität Berlin, Strasse des 17. Juni 135, 10623 Berlin, Germany

**Keywords:** Gender discrimination, Maternal mortality, Sub-Saharan africa, Ecological study

## Abstract

**Background:**

Sub-Saharan Africa suffers from the highest maternal mortality ratio (MMR) in the world, with 542 deaths per 100,000 live births in 2017, relative to a global ratio of 211. Reducing gender-based discrimination (GBD) and increasing the empowerment of women and girls have recently been recognized as prerequisites for improving maternal health. Previous studies have shown GBD to result in low utilization of maternal health services and poorer quality of care. However, limited research is available on the relationship between GBD and maternal mortality in Sub-Saharan Africa (SSA). Therefore, the objective of this study was to assess whether GBD is associated with maternal mortality in SSA.

**Methods:**

We investigated the association between self-reported GBD and maternal mortality in an ecological study. We used data from two surveys: the Demographic and Health Surveys (DHS) and the Afrobarometer. Data refer to 78 sub-national regions, located in nine Sub-Saharan African countries (Benin, Malawi, Mali, Nigeria, Senegal, South Africa, Uganda, Zambia, and Zimbabwe). Data were analyzed using a two-level linear regression model with random intercept. The regression controlled for covariates at region- and country-level.

**Results:**

The proportion of women who reported experiencing GBD varied between 0% in several regions in Benin, Mali, Senegal, South Africa, and Zimbabwe and 24·7% in Atacora, Benin. We identified a positive association between the proportion of women who reported experiencing GBD in a region in the past year and MMR (β 0.88, CI [0.65; 1.12]). A 1% increase in the proportion of women experiencing GBD resulted in an increase of the MMR by nearly two, meaning, an additional two more maternal deaths per 100,000 live births. This association was even more pronounced after adjusting for region-level covariates, but did not change with the inclusion of country-level covariates (β 1.95, CI [1.71; 2.19]).

**Conclusions:**

The study’s findings show that the rate of self-reported GBD is associated with maternal mortality in a region, even after controlling for other factors that are known to influence maternal deaths. However, our model does not rule out endogeneity. Further research is needed to unravel causal pathways between GBD and maternal mortality.

**Supplementary Information:**

The online version contains supplementary material available at 10.1186/s12889-025-24861-z.

## Background

Maternal mortality has declined substantially in the recent past, yet Sub-Saharan Africa (SSA) continues to suffer from the highest maternal mortality ratio (MMR) in the world, with 542 deaths per 100,000 live births in 2017, relative to the global MMR of 211 [1]. In 2015, the United Nations General Assembly adopted the 17 Sustainable Development Goals (SDGs). Target 3·1 of the SDGs calls for a reduction of the global MMR to less than 70 per 100,000 live births by 2030 [2]. The main causes for maternal deaths are obstructed labor, hemorrhage, eclampsia, sepsis, and unsafe abortions [3, 4]. Most of these deaths could be avoided if all women had timely access to high quality maternal health care services [5]. 

Traditionally, strategies to reduce maternal deaths have focused on improving access to high quality reproductive health services. The Millennium Development Goals for instance, through target 5·B sought to improve access to reproductive health services [6]. However, more recently, reducing gender inequality and increasing the empowerment of women and girls have been recognized as prerequisites for improving maternal health [7]. Gender inequality is the systemic disparity in rights, opportunities, and outcomes between genders. Gender inequality is often driven and perpetuated by discrimination, particularly gender-based discrimination [8, 9]. 

In this work, we conceptualize Gender-based discrimination (GBD) as any unequal treatment that occurs because of a person’s gender [10, 11]. GBD can either hinder women’s demand for health care or health workers’ supply of care. Some studies have shown GBD to be related to low utilization of maternal health services [12]. For example, due to culturally entrenched and religiously accepted gender roles and norms, women tend to have lower decision-making power, with negative effects on their ability to access maternal health care services [13], notably when the husband denies permission [5]. Similarly, intimate partner violence can discourage women from seeking family planning services or antenatal care [14]. Discrimination also takes place at health facilities, where women may be denied family planning services if they come alone or if they are unmarried [15]. Male-dominated intra-household resource allocation leads to a lack of resources for women to receive family planning services [13, 16]. More generally, GBD and unequal power balance between men and women are some of the root causes of gender inequities in physical and mental health outcomes, and among the most influential of the social determinants of health [17–20]. GBD also permeates content and process of health research, participation in medical trials, and therefore also medical treatment. Finally, gender imbalances exist among health providers, where women are less present and receive lower payment [21, 22]. The first step to mitigating these unwanted behaviors is increasing awareness of GBD among women.

The Partnership for Maternal, Newborn, and Child Health (PMNCH) explicitly recognizes the need to tackle gender inequality operating at the broader societal level as a means of improving maternal health outcomes [23]. Many studies have assessed the relation between GBD and maternal health care outcomes using different measures and definitions of GBD, such as male-dominated intra-household resource allocation, women’s status, economic dependency, gender inequality, and gender gap [13, 15, 16, 22, 24–30]. Yet, to our knowledge, previous research has not attempted to quantify the relationship between self-reported GBD and maternal mortality in SSA. To address this research gap, we aim to directly investigate whether higher levels of perceived GBD are associated with higher levels of maternal deaths across several SSA countries. More specifically, our study has the following objectives: (1) to map the level of self-reported GBD and MMR in different African countries, (2) to examine the association between self-reported GBD and MMR at the regional level, and (3) to explore the relevance of other regional and country-level factors in explaining MMR.

## Methods

### Study design and data sources

This is an ecological study, with a cross-sectional design. We used data from two main periodic surveys conducted by networks of researchers: Demographic and Health Surveys (DHS) and Afrobarometer.

DHS are large-scale household surveys with an average sample size of 5,000 to 30,000 households, conducted across many low- and middle-income countries (LMICs) using standardized questionnaires [31]. Afrobarometer surveys measure social, political, and economic conditions in more than 30 African countries [32]. Ordinarily, these are measured with the help of face-to-face interviews in applicable local dialects with each survey round containing randomly selected samples of 1,200 or 2,400 people in each country. Working with trained and skilled national partners ensures the quality of data collection [32]. 

We used data from the DHS for our outcome (dependent) variable, i.e., MMR. Data from Afrobarometer was used for our main independent variable, i.e., GBD. Since DHS phase VII, the inclusion of two questions to exclude deaths due to an act of violence or an accident have improved maternal mortality estimates [33]. Therefore, this paper utilized data from DHS phase VII, conducted between 2015 and 2018 (see Appendix 1). From round seven (R7), Afrobarometer started to include a question to explore self-reported GBD of women [34]. Therefore, we used data from Afrobarometer R7, conducted between 2016 and 2018. We decided against using the DHS gender-based violence module to construct our GBD variable since we aimed to investigate and capture GBD in general, not only in its overt manifestation as violence [31]. 

Both data sources provide geocoded data at the region-level. We picked all SSA countries for which we had data from DHS and Afrobarometer for the mentioned time periods and matched the datasets at the corresponding geocoded regional level. That led to the following country selection: Benin, Malawi, Mali, Nigeria, Senegal, South Africa, Uganda, Zambia, and Zimbabwe. We then modeled the analysis as a random intercept two-level model to assess further information at the region- and at the country-level.

## Variables and measurement

### Outcome variable

Our outcome variable of interest is the MMR, which captures the number of maternal deaths per 100,000 live births [35]. To estimate maternal mortality, we built a variable from DHS using the sibling history approach [36]. Using a catalogue of detailed questions, this method asks women to name and count their sisters who died from maternal causes [36]. We coded this variable as continuous, differing by region of residence, and calculated it as follows [36]:

1 $$\:\mathrm{MMR}=\frac{\mathrm{MMRate}}{\mathrm{FR}}\:\mathrm x\:\text{100,000},$$

2 $$\:\mathrm{MMRate}=\frac{\mathrm{Number}\:\mathrm{of}\:\mathrm{maternal}\:\mathrm{deaths}}{\mathrm{Womenyears}\:\mathrm{of}\:\mathrm{exposure}\:\mathrm{of}\:\mathrm{sisters}\:}\mathrm x\text{1,000},$$

3 $$\:\mathrm{FR}=\frac{\mathrm{Number}\:\mathrm{of}\:\mathrm{births}}{\mathrm{Womenyears}\:\mathrm{of}\:\mathrm{exposure}\:\mathrm{of}\:\mathrm{female}\:\mathrm{respondents}}\mathrm x\text{1,000},$$

where MMRate is the maternal mortality rate, and FR is the fertility rate. We used the DHS guide to compute and estimate all relevant statistics with respect to DHS-7. We calculated the number of births and the number of maternal deaths for a period of zero to six years preceding the survey [37]. Women-years of exposure are the sum of women living in a given preceding time period, in our case zero to six years [36]. We generated the number of births out of the DHS Birth’s Recode and the number of maternal deaths as well as the women-years of exposure out of the DHS Individual Recode [38].

### Exposure variable

Our main independent (exposure) variable of interest is the proportion of women who reported having experienced gender-based discrimination in the past year. Afrobarometer asks: “In the past year, how often, if at all, have you personally been discriminated against or harassed based on […] your gender?” [34] with answer categories distinguishing never, once or twice, several times, or many times. We transformed this categorical variable into a binary variable (0 = never/once or twice; 1 = several or many times.) and then used the proportion of women who reported having experienced GBD several or many times out of the full sample of women interviewed in a given region as a continuous variable.

### Control covariates

At both the region- and the country-levels, we controlled for several covariates that have been shown to be associated with maternal mortality in previous analyses [39–46]. At the region-level we integrated the proportion of women who (1) have any school education, (2) are assigned with a high lived poverty index (LPI), which measures how frequently a person goes without basic needs such as food, water, or medicine [47], (3) have difficulties in obtaining medical treatments in general (not only relating to maternity), (4) never had to pay a bribe to obtain medical treatments, and who (5) have access to a piped water system close to their place of residence. At the country-level, we controlled for the (6) UNAIDS’ estimated HIV prevalence among the total population of ages 15–49, (7) the WHO’s calculated current health expenditure per capita in US$, and (8) the adolescents’ fertility rate measured by the United Nations Population Division, which means the average number of births per 1,000 women ages 15–19 (see Tables 1 and 2). We controlled all independent variables for multicollinearity and found that correlation values are consistently below ± 0·73 (>95% are below ± 0·7) (see Appendix 2). We did not control for median age or fertility rates, as these variables are captured by the outcome (dependent) variable, MMR.

**Table 1:** Variables, definitions, measurements, and sources

Variable	Definition	Measurement	Data source and Date
Outcome Variable			
MMR	Number of maternal deaths per 100,000 live births during the six years preceding the survey per region	Continuous Variable	DHS-VII, 2015-2018 Individual and Birth Recode [48]
Exposure Variable (Main Independent Variable)			
Self-reported experiencing GBD	Proportion of women reporting experiencing gender-based discrimination in the year preceding the survey per region	Continuous Variable, Unit of Measure in %	Afrobarometer R7, 2016–2018 [34]
Covariates region-level			
School education	Proportion of women who have any kind of school education	Continuous Variable, Unit of Measure in %	DHS-VII, 2015-2018 Individual and Birth Recode [48]
High LPI	Proportion of women who are assigned with a high lived poverty index	Continuous Variable, Unit of Measure in %	Afrobarometer R7, 2016–2018 [34]
Difficulties in obtaining medical treatment	Proportion of women who have difficulties in obtaining medical treatments	Continuous Variable, Unit of Measure in %	Afrobarometer R7, 2016–2018 [34]
Never pay bribes for medical treatment	Proportion of women who never had to pay a bribe to obtain medical treatment	Continuous Variable, Unit of Measure in %	Afrobarometer R7, 2016–2018 [34]
Access to water	Proportion of women who have access to a piped water system	Continuous Variable, Unit of Measure in %	Afrobarometer R7, 2016–2018 [34]
Covariates country-level			
HIV Prevalence	HIV Prevalence among the population of ages 15–49	Continuous Variable, Unit of Measure in %	UNAIDS, 2020 [49]
Health Expenditure	Current health expenditure per capita	Continuous Variable, Unit of Measure in US$	WHO, 2018 [50]
Adolescents’ Fertility Rate	Average number of births per 1,000 women ages 15–19	Continuous Variable, Unit of Measure in %	UNPD, 2019 [51]

## Analytical approach

**Fig. 1:**
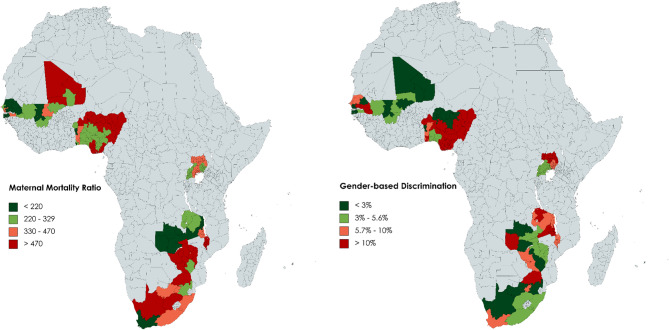
Mapped levels of MMR and reported GBD per region

To generate an initial overview, we first mapped the levels of self-reported GBD and MMR in the selected 78 regions across the nine countries at a region-level. We classified four subgroups for each of the two continuous variables, from low, over medium-low, and medium-high, to high levels. For self-reported GBD the subgroups range from ‘<3%’, over ‘3–5·6%’, ‘5·7–10%’ to ‘>10%’. We formed these groups tailored to the distribution of the values, so that every group would contain approximately the same number of regions. We contrasted both variables with the help of a figure showing the levels in colors (see Fig. 1). For instance, in Mopti, Mali, we found, based on the Afrobarometer data, that 1·5% of the women reported experiencing GBD. Mopti belongs to the lowest level of < 3% reported GBD and that is why Mopti is colored in dark green in Fig. 1.

To examine the association between MMR and self-reported GBD and to assess the relevance of other region- and country-level factors in explaining MMR, we conducted a random intercept two-level model using the continuous variable measures in a linear regression. Level 1 is the region of residence, which is nested in level 2, the country. For this analysis, we used data from 160,275 women from the DHS survey and from 5,928 women from the Afrobarometer survey, living in $\:{n}_{i}=78\:\text{r}\text{e}\text{g}\text{i}\text{o}\text{n}\text{s}$ (level 1), nested in $\:{n}_{j}=9\:\text{c}\text{o}\text{u}\text{n}\text{t}\text{r}\text{i}\text{e}\text{s}$ (level 2). Since this in an ecological analysis, the model worked with the regional proportion of experienced GBD (based on the Afrobarometer), and the data were merged at the regional level – not at the level of individual cases. Choosing a random-intercept model, we interpreted the slope, i.e., the coefficients of the independent variables, as fixed across all 78 regions. On the other hand, the intercept is random across all 78 regions due to region- and country-specific residuals. Our analysis consists of four different models, each adjusted for more covariates. First, we conducted the Null Model, which only concentrates on our outcome variable and gives out the overall mean of the MMR across all regions. Then we integrated step by step our main independent variable of interest (Model 1), all region-level covariates (Model 2), and all country-level covariates (Model 3). By adjusting the model for independent variables, the model produces coefficients that show the association between the independent variables and the MMR, and show the independent association of self-reported GBD with MMR. We conducted all analyses of our random intercept two-level model in the statistics software SPSS (PASW Statistics 18.0). The equation of the full model (Model 3) is:

4 $$\begin{aligned}\:{\mathrm y}_{\mathrm{ij}}&={\mathrm\beta\:}_0+{\mathrm\beta\:}_1{\mathrm x}_{1\mathrm{ij}}+{\mathrm\beta\:}_2{\mathrm x}_{2\mathrm{ij}}+{\mathrm\beta\:}_3{\mathrm x}_{3\mathrm{ij}}\\&+{\mathrm\beta\:}_4{\mathrm x}_{4\mathrm{ij}}+{\mathrm\beta\:}_5{\mathrm x}_{5\mathrm{ij}}+{\mathrm\beta\:}_6{\mathrm x}_{6\mathrm{ij}}+{\mathrm\beta}_7{\mathrm x}_7\mathrm j\\&+{\mathrm\beta}_8{\mathrm x}_8\mathrm j+{\mathrm\beta}_9{\mathrm x}_9\mathrm j+{\mathrm u}_{\mathrm j}+{\mathrm e}_{\mathrm i}\mathrm j,\end{aligned}$$

where $\:{y}_{ij}$ represents the MMR outcome variable for region $\:i$ in country $\:j$. Every $\:x$ represents a predictor variable, $\:{\beta\:}_{0}$ is the overall mean of the MMR across all 78 regions, $\:{\beta\:}_{1-9}$ are the described coefficients or fixed effects of the predictor variables on the MMR, and $\:{e}_{ij}$ and $\:{u}_{j}$ are the region- and country-specific residuals. Besides the estimates of fixed parameters as well as individual residuals, each model produces the Akaike’s Information Criterion (AIC). The comparison of the AIC for each model shows whether the inclusion of the covariates improves the fit of the model or not, i.e., if the AIC is smaller than the AIC of the model conducted before, it is a fit improvement [52]. Click or tap here to enter text.

## Results

Table 2 describes the full sample population and distribution of the answers by variable used in the regression models. The majority of women (93%) did not experience gender discrimination in the last year, although there is high variance across countries and sub-regions (Fig. 1). Most women have no school education (72%) and don’t have piped water at home (54%).

**Table 2:** Descriptive statistics of regional independent variables

	Study Population (*n* = 160,275 [DHS]/5,928 [Afrobarometer)	
		%
Experienced gender discrimination – total	5925	100
Yes	427	7
No	5489	93
Have school education – total	160,275	100
Yes	44,893	28
No	115,382	72
High Lived Poverty Index (LPI) – total	5884	100
Yes	1024	17
No	4860	83
Obtaining medical treatment – total	5927	100
Difficult	1772	30
Easy	2080	35
No contact	2069	35
Having to pay a bribe to obtain medical treatment – total	5927	100
Never	3455	58
At least once	397	7
No contact	2069	35
Have a piped water system in primary sampling unit/enumeration area – total	5928	100
Yes	2674	45
No	3225	54
*Sum of counts for each variable may not add up to total due to missing data		

The two maps in Fig. 1 provide an overview of the levels of MMR and self-reported GBD across regions. They show that the reported experience of GBD is highest in Benin, Atacora (24·7%), Senegal, Tambacounda (23·0%), and Zambia, Northern region (18·2%). The highest MMRs, i.e., the number of maternal deaths per 100,000 live births, are in South Africa, Northern Cape (2079), Mali, Kidal (1992), and Mali, Toumbouctou (1273) (see Appendices 3, 4). Notably, large variations of both GBD and MMR were depicted among selected regions at in-country level in SSA.

Pooling data across 78 regions, located in nine countries, the Null Model generates an overall estimate of 417 (95% CI 310–523), indicating 417 maternal deaths per 100,000 live births (see Table 3). In Model 1, adjusted only for the main independent variable of interest, the coefficient of 0·88 (95% CI 0·65 − 1·12) indicates that a 1% increase in the proportion of women reporting GBD results in nearly one more maternal death per 100,000 live births. Adjusting for region-level covariates (Model 2), we observe an even more pronounced association between self-reported GBD and MMR, with the coefficient standing at 1·95 (95% CI 1·71 − 2·19), and this does not change with the inclusion of country-level covariates (Model 3). In the full model (Model 3), coefficients of most covariates point in the expected directions, which are positive for LPI (4·59 (95% CI 4·45 − 4·72)), difficulty in obtaining medical treatment (0·42 (95% CI 0·26 − 0·58)), HIV prevalence (38·15 (95% CI 13·67 − 62·62)), and adolescents’ fertility rate (0·92 (95% CI −2·8 − 4·64)); and negative for school education (−6·05 (95% CI −6·24-(−5·96))), never paying bribes for medical treatments (−1·74 (95% CI −1·88-(−1·58))), and health expenditure (−0·24 (95% CI −1·36 − 0·89)). However, the coefficient of access to piped water is positively associated with higher MMR (1·59 (95% CI 1·53 − 1·65)).

Looking at the AIC, the inclusion of the regional covariates improved the fit of Model 2 compared with the Null Model. By adding the country-level covariates, the AIC remained nearly the same (see Table 3).

**Table 3:** Results of the two-level linear regression analysis

	Null Model		Model 1 - adjusted for main independent variable		Model 2- adjusted for covariates at region-level		Model 3 - adjusted for covariates at country-level	
	Fixed Effects	95% CI	Fixed Effects	95% CI	Fixed Effects	95% CI	Fixed Effects	95% CI
MMR	417*	(310; 523)	410*	(303; 517)	781*	(597; 965)	451*	(14; 889)
Reported GBD			0.88*	(0.65; 1.12)	1.95*	(1.71; 2.19)	1.95*	(1.71; 2.19)
School education					−6.05*	(−6.14; −5.95)	−6.05*	(−6.24; −5.96)
High LPI					4.59*	(4.45; 4.72)	4.59*	(4.45; 4.72)
Difficulty in obtaining medical treatment					0.42*	(0.26; 0,58)	0.42*	(0.26; 0.58)
Never pay bribes for medical treatment					−1.73*	(−1.88; −1.58)	−1.74*	(−1.88; −1.58)
Access to water					1.59*	(1.53; 1,65)	1.59*	(1.53; 1.65)
HIV Prevalence							38.15*	(13.67; 62.62)
Health Expenditure							**−0.24**	(−1.36; 0.89)
Adolescents’ Fertility Rate							**0.92**	(−2.8; 4.64)
Akaike’s information criterion (AIC); % change to null model	2217826,771		2217774,238; −0.002%		2187952,673; −1.35%		2187947,210; −1.35%	
*statistically significant at α = 0.05								

## Discussion

This is the first study to investigate the association between the proportion of women reporting GBD and MMR across several countries in SSA. Since data on obstetrics and MMR is usually incomplete or not routinely collected [53, 54], our work used alternative data sources based on surveys to monitor MMR and GBD. In addition, we built on standardized data collected across countries and regions, allowing for cross-country comparison.

We found a significant positive association between self-reported GBD and the number of maternal deaths, with nearly two more deaths per 1% increase in the proportion of women reporting being discriminated. There was large variation in both self-reported GBD and MMR across regions within countries, which requires a better understanding of the underlying factors.

Several previous studies have focused on GBD in the context of specific aspects of maternal health, such as the association between gender inequality and low utilization of maternal health services [12, 24, 26]. In fact, GBD is increasingly being recognized as affecting women’s health, by shaping resource allocation, both within and outside the household, and limiting access to health services [55, 56]. However, our analysis is the first to show that self-reported GBD is independently associated with MMR. In our model, the effect size of self-reported GBD was similar or larger to that of many factors that are well-known for their association with maternal mortality, such as having access to water, never having paid bribes, or the level of national health expenditures, even if it was considerably smaller than the effect sizes of education, or general poverty [39, 40, 45]. 

Our findings expand the literature by identifying GBD as an important risk factor to MMR that is related to the society where women live. More well known individual level risk factors include women’s characteristics such as age (younger than 20 or older than 35), weight (under or over), nutritional status, anemia, comorbidities, and low education; pregnancy characteristics such as number of pregnancies, having twins, pregnancy complications, and size of fetus; characteristics of the event of the delivery such as obstructed birth, Caesarean mode of delivery, the absence of a health professional at birth; and low access to maternal care such as antenatal care or postpartum care [53, 57–61]. However, the experience of GBD affects all women in a given region, and therefore has a pervasive effect on women’s health. Other studies found that low education of the partner is also a risk factor to MMR [61], yet it is still an individual level risk factor. Our study adds a collective risk factor that affects a whole society, which in the context of LMICs goes beyond the lack of resources or poverty.

Several studies have shown a negative association between GBD and women’s health beyond MMR. GBD was found to exacerbate the burden of chronic diseases, as it increased substantially the risk of having at least one physical health condition [62]. Perceived GBD negatively affects not only physical but also mental health and wellbeing [63, 64], while reducing access to women’s health care [65]. Even if women might have access to health care, GBD can prevent women from seeking antenatal and maternal health services due to gender inequalities in the quality of treatments [15]. GBD is expressed by women’s lower autonomy [66] and is associated with poorer mental and physical health and higher mortality not only for women, but for their children too [67]. While perceived GBD is more reported among women with high education or high SES, this group is also more resilient to the negative spillover effects of GBD on health [63, 68].

## Strengths and limitations

The strengths of our paper are that we showed a positive association between GBD and maternal mortality based on standardized data collected across countries and regions. While there is evidence on the negative association of GBD and women’s health, the quality of these studies is not high [67]. Our work fills this gap by providing high-quality evidence on the increased burden of GBD on maternal health, specifically on MMR. Moreover, due to the usage of data collected across regions, our findings allow for cross-country comparison.

Our study has several limitations. First, our main outcome variable of regional MMR suffers from the problem that DHS does not ask for the residence of the women who died but instead assigns the maternal deaths to the regions the interviewed sisters live in, although the place of residence of the sisters (the one who died and the one who is interviewed) is not necessarily the same [36]. Also, some of our DHS estimates for the MMR differ significantly from estimates made by the WHO, UNICEF or other data sources [75]. That is why, there might be discrepancies between the MMR values used for our analysis and preceding findings made by other researchers. For instance, the overall MMR in South Africa, i.e., 532, is outstandingly high in our model compared to other data sources and estimation methods (see Appendix 4) [76]. More realistic estimates of MMR than those made in this analysis could lead to different correlations between MMR and self-reported GBD. Second, our main independent variable is a subjective and likely imprecise measure of experiencing GBD because the Afrobarometer asks only a single question to explore a woman’s experience with GBD [34]. As the understanding of GBD varies across countries, regions or individuals, our self-reported GBD variable reflects subjective realities, awareness, cultural norms and expectations, with minorities underestimating its frequency [77]. While there are indicators that measure gender equality in different fields such as education, health, labor market and political life [77–83], no indicator directly measures gender discrimination. The prevalence, or an ‘objective’ magnitude of GBD remains unknown. In addition, GBD has many dimensions, and while the Afrobarometer self-reported GBD question is broad and attempts to capture the overall experience of GBD, it does not indicate the source or context of GBD. For example, if it is domestic abuse or economic dependency that hinders women’s demand for health care, or GBD at the supply side, resulting in poor quality of care. More specific questions about the various dimensions of GBD could render refined results to support policy that tackles GBD. However, to our knowledge, there is no cross-country survey that explores the different dimensions of GBD in SSA. Third, data was unavailable across countries for several indicators that have been previously found to be associated with maternal mortality, such as health insurance coverage, access to or the quality of maternal care, chronic diseases or comorbidities [26, 84]. This means that our model does not fully account for all factors that influence maternal mortality, even though it does control for some of the most important ones [39–46]. In addition, we did not include unmet need for family planning such as reproductive health services and use of contraceptives as a control variable because of its strong correlation with self-reported GBD [15, 42, 85]. It is important to note that as an ecological study, we found an association between proportions of women who reported having experienced GBD and the regional MMR, but we did not investigate the relationship between a single woman being exposed to discrimination and the probability of her dying due to maternal health causes. Finally, we could not rule out endogeneity, as the same set of independent variables that are responsible for self-reported GBD may also be responsible for MMR [86]. 

### Policy implications and future research

Our study has important implications for both policy and future research. First, our findings suggest that addressing GBD could contribute both to reducing MMR and to achieving the SDGs [2]. UNICEF’s “Gender Action Plan 2022–2025” provides an example of a high-level strategy that aims to address gender equality and reduce GBD [69]. National health strategies could follow this example and incorporate measures aimed at reducing GBD – not only because of its role in explaining maternal mortality. In addition, given that disrespectful maternal health care provision at health facilities has been widely reported in SSA, targeted training of health care providers on topics such as GBD and gender-based violence could contribute to improving the situation [13, 15, 16, 70]. 

Second, the findings of our study and its limitations point to the importance of better understanding and measuring the different dimensions and sources of GBD. While existing research has focused on associations between single dimensions of GBD, such as male-dominated intra-household resource allocation, and maternal health care outcomes [13, 16], limited evidence is available on the multidimensional effects of GBD on maternal health. One reason for this lack of a comprehensive understanding of the effects of GBD on maternal health is that a reliable tool for measuring the multiple dimensions of GBD is currently unavailable. The development of a comprehensive standard questionnaire on GBD could potentially overcome this problem and contribute to a better assessment of GBD across different cultural and educational backgrounds.

Third, while there is a rich body of evidence on risk factors associated with maternal mortality [58, 61, 71], more research is needed that explores the causal pathways linking GBD with maternal health outcomes, including maternal mortality. These pathways may include external mechanisms, such as male- dominated decision-making spaces leading to inadequate consideration of women’s health needs in the development of national health strategies and policies; [13] or the negative impact of GBD on women’s education and financial resources, which hampers their ability to use timely and appropriate health services [13, 16, 22, 27, 30]. Pathways may also include internal mechanisms, as women could internalize the symbolic societal violence generated by GBD, and as a consequence start to under-value their lives and limit their self-efficacy to take action to prevent maternal mortality [72]. This may disempower women and affect how they perceive the value of their own lives [73, 74]. In addition, more research is needed to explore perceived GBD in healthcare settings, assessing the effects of undervaluation and different treatment of women by health providers, and its impact on MMR. Finally, more knowledge could be produced on the effects of gender imbalance of health research and treatment guidelines and MMR [19]. 

## Conclusions

In conclusion, our study found that maternal mortality is associated with self-reported GBD even after controlling for other determinants of maternal mortality. Our findings are important because they suggest that actions and measures to address GBD could help to improve maternal health and reduce maternal mortality. Further research is needed to better understand the different dimensions and sources of GBD as well as to unravel causal pathways between GBD and maternal health outcomes. A better understanding of GBD is a prerequisite for designing effective interventions to address the different sources of GBD. Ultimately, addressing GBD is needed not only because of its detrimental effects on maternal health but also to achieve the broader societal goals of gender equality.

## Supplementary Information

Supplementary material 1.

## Data Availability

The datasets analysed during the current study are available in the Demographic and Health Surveys (DHS) https://dhsprogram.com/publications/publication-dhsq7-dhs-questionnaires-and-manuals.cfm and Afrobarometer repositories https://www.afrobarometer.org/data/.

## Abbreviations

AIC: Akaike’s information criterion
CI: Confidence interval
DHS: Demographic and health surveys
FR: Fertility rate
GBD: Gender-based discrimination
HIV: Human immunodeficiency virus
LMICs: Low- and middle-income countries
LPI: Lived poverty index
MMR: Maternal mortality ratio
PMNCH: The partnership for maternal, newborn, and child health
R7: Round seven
SSA: Sub-Saharan Africa

## References

[1] Organization WH. Trends in maternal mortality 2000 to 2017: estimates by WHO, UNICEF, UNFPA, world bank group and the united nations population division: executive summary. World Health Organization; 2019.

[2] United Nations General Assembly. A/RES/70/1. Transforming our world: the 2030 agenda for sustainable development. United Nationa General Assembly; 2015.

[3] WHO. The World Health Report. 2005. Make every mother and child count - Health and Education Advice and Resource Team. Geneva: World Health Organization; 2005.

[4] Kassebaum NJ, Barber RM, Dandona L, Hay SI, Larson HJ, Lim SS, et al. Global, regional, and National levels of maternal mortality, 1990–2015: a systematic analysis for the global burden of disease study 2015. Lancet. 2016;388:1775–812. 10.1016/S0140-6736(16)31470-2. 27733286 10.1016/S0140-6736(16)31470-2 PMC5224694

[5] Kyei-Nimakoh M, Carolan-Olah M, McCann TV. Access barriers to obstetric care at health facilities in sub-Saharan Africa-a systematic review. Syst Rev. 2017;6. 10.1186/S13643-017-0503-X. 10.1186/s13643-017-0503-x PMC5461715 28587676

[6] United Nations. Goal 5: Improve Maternal Health. United Nations Millennium Development Goals. 2021. https://www.un.org/millenniumgoals/maternal.shtml. Accessed 28 Jan 2023.

[7] Organization WH. Strategies towards ending preventable maternal mortality (EPMM). World Health Organization; 2015.

[8] Beugelsdijk S, Gelfand MJ, Kleinhempel J. Evolving patterns of gender inequality over time and across countries: new theoretical perspectives and an emerging research agenda. J Int Bus Stud. 2025. 10.1057/s41267-025-00799-7.

[9] Jayachandran S. The roots of gender inequality in developing countries. Annu Rev Econ. 2015;7:63–88. 10.1146/annurev-economics-080614-115404.

[10] de la Torre-Pérez L, Oliver-Parra A, Torres X, Bertran MJ. How do we measure gender discrimination? Proposing a construct of gender discrimination through a systematic scoping review. Int J Equity Health. 2022;21:1–11. 10.1186/S12939-021-01581-5/FIGURES/2. 34980116 10.1186/s12939-021-01581-5 PMC8722302

[11] Salvini S. Gender discrimination. In: Michalos AC, editor. Encyclopedia of quality of life and Well-Being research. Dordrecht: Springer Netherlands; 2014. pp. 2424–7. 10.1007/978-94-007-0753-5_1126.

[12] Ahmed S, Creanga AA, Gillespie DG, Tsui AO. Economic Status, education and empowerment: implications for maternal health service utilization in developing countries. PLoS ONE. 2010;5:1–6. 10.1371/journal.pone.0011190. 10.1371/journal.pone.0011190 PMC2890410 20585646

[13] Morgan R, Tetui M, Kananura RM, Ekirapa-Kiracho E, George AS. Gender dynamics affecting maternal health and health care access and use in Uganda. Health Policy Plan. 2017;32 suppl5:v13–21. 10.1093/HEAPOL/CZX011. 29244103 10.1093/heapol/czx011 PMC5886085

[14] Alhusen JL, Ray E, Sharps P, Bullock L. Intimate Partner Violence During Pregnancy: Maternal and Neonatal Outcomes. https://home.liebertpub.com/jwh. 2015;24:100–6. 10.1089/JWH.2014.4872 10.1089/jwh.2014.4872 PMC4361157 25265285

[15] Oduenyi C, Banerjee J, Adetiloye O, Rawlins B, Okoli U, Orji B, et al. Gender discrimination as a barrier to high-quality maternal and newborn health care in nigeria: findings from a cross-sectional quality of care assessment. BMC Health Serv Res. 2021;21:198. 10.1186/s12913-021-06204-x. 33663499 10.1186/s12913-021-06204-x PMC7934485

[16] Irving M, Kingdon GG. Gender patterns in household health expenditure allocation: a study of South Africa. 2008.

[17] Miani C, Wandschneider L, Niemann J, Batram-Zantvoort S, Razum O. Measurement of gender as a social determinant of health in epidemiology—A scoping review. PLoS ONE. 2021;16:e0259223. 10.1371/JOURNAL.PONE.0259223. 34731177 10.1371/journal.pone.0259223 PMC8565751

[18] Pedrana L, Pamponet M, Walker R, Costa F, Rasella D. Global Health Action Scoping review: national monitoring frameworks for social determinants of health and health equity Scoping review: national monitoring frameworks for social determinants of health and health equity. 2016. 10.3402/gha.v9.28831 10.3402/gha.v9.28831 PMC4744868 26853896

[19] Sen G, Östlin P. Gender as a social determinant of health: Evidence, policies, and innovations. In: Gender Equity in Health: The Shifting Frontiers of Evidence and Action. Taylor and Francis; 2009. p. 1–46. 10.4324/9780203866900-9/GENDER-SOCIAL-DETERMINANT-HEALTH-EVIDENCE-POLICIES-INNOVATIONS-GITA-SEN-PIROSKA-.

[20] Borrell C, Artazcoz L, Gil-González D, Pérez G, Rohlfs I, Pérez K. Perceived sexism as a health determinant in Spain. https://home.liebertpub.com/jwh. 2010;19:741–50. 10.1089/JWH.2009.1594 10.1089/jwh.2009.1594 20350207

[21] Van Gijsbers CMT, Van Vliet KP, Kolk AM. Gender perspectives and quality of care: towards appropriate and adequate health care for women. Soc Sci Med. 1996;43:707–20. 10.1016/0277-9536(96)00115-3. 8870135 10.1016/0277-9536(96)00115-3

[22] Govender V, Penn-Kekana L. Gender biases and discrimination: a review of health care interpersonal interactions. Glob Public Health. 2008;3 SUPPL. 1:90–103. 10.1080/17441690801892208 10.1080/17441690801892208 19288345

[23] PMNCH. PMNCH - Partnership for Maternal, Newborn and Child Health. 2023. https://pmnch.who.int/. Accessed 29 Jan 2023.

[24] Chirowa F, Atwood S, van der Putten M. Gender inequality, health expenditure and maternal mortality in sub-Saharan africa: A secondary data analysis. Afr J Prim Health Care Fam Med. 2013;5. 10.4102/PHCFM.V5I1.471.

[25] Choe S-A, Cho S, Kim H. Gender gap matters in maternal mortality in low and lower-middle-income countries: A study of the global gender gap index. Glob Public Health. 2017;12:1065–76. 10.1080/17441692.2016.1162318. 27021475 10.1080/17441692.2016.1162318

[26] Chukuezi C. Socio-cultural factors associated with maternal mortality in Nigeria. Res J Soc Sci. 2010;1:22–6.

[27] Shen C, Williamson JB. Maternal mortality, women’s status, and economic dependency in less developed countries: a cross-national analysis. Soc Sci Med. 1999;49:197–214. 10.1016/S0277-9536(99)00112-4. 10414829 10.1016/s0277-9536(99)00112-4

[28] Lan CW, Tavrow P. Composite measures of women’s empowerment and their association with maternal mortality in low-income countries. BMC Pregnancy Childbirth. 2017;17:1–11. 10.1186/S12884-017-1492-4/TABLES/6. 29143614 10.1186/s12884-017-1492-4 PMC5688512

[29] Matthew O, Adeniji A, Osabohien R, Olawande T, Atolagbe T, Gender Inequality. Maternal mortality and inclusive growth in Nigeria. Soc Indic Res. 2020;147:763–80. 10.1007/S11205-019-02185-X/TABLES/9.

[30] King TL, Kavanagh A, Scovelle AJ, Milner A. Associations between gender equality and health: a systematic review. Health Promot Int. 2020;35:27–41. 10.1093/HEAPRO/DAY093. 31916577 10.1093/heapro/day093

[31] DHS Program. Demographic and Health Surveys - Data. 2023. https://dhsprogram.com/Data/. Accessed 28 Jan 2023.

[32] Afrobarometer. Surveys and methods – Afrobarometer. 2023. https://www.afrobarometer.org/surveys-and-methods/. Accessed 28 Jan 2023.

[33] DHS Program. Adult and Maternal Mortality Module Model Woman’s Questionnaire. 2016.

[34] Afrobarometer. Analyse online – Afrobarometer. 2023. https://www.afrobarometer.org/online-data-analysis/. Accessed 28 Jan 2023.

[35] World Health Organization. Maternal mortality ratio (per 100 000 live births). The Global Health Observatory. 2023. https://www.who.int/data/gho/indicator-metadata-registry/imr-details/26. Accessed 28 Jan 2023.

[36] Croft, Trevor N, Marshall AMJ, Allen C, et al. Guide to DHS statistics DHS-7 (version 2). Demographic and Health Surveys Program. Rockville; 2020.

[37] DHS Program. Maternal Mortality and Pregnancy-related Mortality. 2018. https://dhsprogram.com/data/Guide-to-DHS-Statistics/Maternal_Mortality_and_Pregnancy-related_Mortality.htm. Accessed 28 Jan 2023.

[38] DHS Program. The DHS Program - Dataset Types. 2023. https://dhsprogram.com/data/dataset-types.cfm. Accessed 29 Jan 2023.

[39] Batist J. An intersectional analysis of maternal mortality in Sub-Saharan africa: a human rights issue. J Glob Health. 2019;9. 10.7189/JOGH.09.010320. 10.7189/jogh.09.010320 PMC6551548 31217956

[40] Lanre-Abass BA. Poverty and maternal mortality in nigeria: towards a more viable ethics of modern medical practice. Int J Equity Health. 2008. 10.1186/1475-9276-7-11. 7. 18447920 10.1186/1475-9276-7-11 PMC2390565

[41] Sumankuuro J, Crockett J, Wang S. Perceived barriers to maternal and newborn health services delivery: a qualitative study of health workers and community members in low and middle-income settings. BMJ Open. 2018;8. 10.1136/BMJOPEN-2017-021223. 10.1136/bmjopen-2017-021223 PMC6231574 30413495

[42] Ruiz-Cantero MT, Guijarro-Garvi M, Bean DR, Martínez-Riera JR, Fernández-Sáez J. Governance commitment to reduce maternal mortality. A political determinant beyond the wealth of the countries. Health Place. 2019;57:313–20. 10.1016/J.HEALTHPLACE.2019.05.012. 31146194 10.1016/j.healthplace.2019.05.012 PMC6873917

[43] Cheng JJ, Schuster-Wallace CJ, Watt S, Newbold BK, Mente A. An ecological quantification of the relationships between water, sanitation and infant, child, and maternal mortality. Environ Health. 2012;11:4. 10.1186/1476-069X-11-4. 22280473 10.1186/1476-069X-11-4 PMC3293047

[44] Moran NF, Moodley J. The effect of HIV infection on maternal health and mortality. Int J Gynecol Obstet. 2012. 10.1016/J.IJGO.2012.03.011. 119 SUPPL.1. 10.1016/j.ijgo.2012.03.011 22889550

[45] Buor D, Bream K. An analysis of the determinants of maternal mortality in Sub-Saharan Africa. J Womens Health. 2004;13:926–38. 10.1089/jwh.2004.13.926. 10.1089/jwh.2004.13.926 15671708

[46] World Health Organization. The Second decade: improving adolescent health and development. 2001.

[47] Mattes R, Dulani B, Gyimah-Boadi E. Africa’s growth dividend? Lived poverty drops across much of the continent. 2016.

[48] DHS Program. The DHS Program - Available Datasets. https://dhsprogram.com/data/available-datasets.cfm. Accessed 30 Jan 2023.

[49] UNAIDS data 2020. Report https://www.unaids.org/sites/default/files/media_asset/2020_aids-data-book_en.pdf

[50] WHO. Global Health Expenditure Database. 2023. https://apps.who.int/nha/database. Accessed 30 Jan 2023.

[51] UNDP. Human development report 2021-22. United Nations Development Programme; 2022.

[52] Leckie G. Cross-classified multilevel models. 2019.

[53] Bauserman M, Thorsten VR, Nolen TL, Patterson J, Lokangaka A, Tshefu A, et al. Maternal mortality in six low and lower-middle income countries from 2010 to 2018: risk factors and trends. Reprod Health. 2020;17:173. 10.1186/s12978-020-00990-z. 33334343 10.1186/s12978-020-00990-z PMC7745363

[54] Bola R, Ngonzi J, Ujoh F, Kihumuro RB, Lett R. An evaluation of obstetrical data collection at health institutions in Mbarara Region, Uganda and Benue State, Nigeria. Pan Afr Med J. 2024;47:109. 10.11604/pamj.2024.47.109.36295. 38766561 10.11604/pamj.2024.47.109.36295 PMC11101309

[55] Garrison-Desany HM, Wilson E, Munos M, Sawadogo-Lewis T, Maïga A, Ako O, et al. The role of gender power relations on women’s health outcomes: evidence from a maternal health coverage survey in Simiyu region, Tanzania. BMC Public Health. 2021;21:909. 10.1186/s12889-021-10972-w. 33980197 10.1186/s12889-021-10972-w PMC8117490

[56] Azad AD, Charles AG, Ding Q, Trickey AW, Wren SM. The gender gap and healthcare: associations between gender roles and factors affecting healthcare access in central Malawi, June–August 2017. Arch Public Health. 2020;78:119. 10.1186/s13690-020-00497-w. 33292511 10.1186/s13690-020-00497-w PMC7672876

[57] Bola R, Ujoh F, Ukah UV, Lett R. Assessment and validation of the community maternal danger score algorithm. Glob Health Res Policy. 2022;7:6. 10.1186/s41256-022-00240-8. 35148791 10.1186/s41256-022-00240-8 PMC8832636

[58] Diana S, Wahyuni CU, Prasetyo B. Maternal complications and risk factors for mortality. J Public Health Res. 2020;9. 10.4081/jphr.2020.1842. :jphr.2020.1842. 10.4081/jphr.2020.1842 PMC7376486 32728581

[59] Symonds NE, Vidler M, Wiens MO, Omar S, English LL, Ukah UV, et al. Risk factors for postpartum maternal mortality and hospital readmission in low- and middle-income countries: a systematic review. BMC Pregnancy Childbirth. 2023;23:303. 10.1186/s12884-023-05459-y. 37120529 10.1186/s12884-023-05459-y PMC10148415

[60] Nik Hazlina NH, Norhayati MN, Shaiful Bahari I, Mohamed Kamil HR. The prevalence and risk factors for severe maternal morbidities: A systematic review and Meta-Analysis. Front Med. 2022;9. 10.3389/fmed.2022.861028. 10.3389/fmed.2022.861028 PMC8968119 35372381

[61] Kolleh EM, Bestman PL, Bajinka O, Weamie JYS, Luo J. Maternal mortality and its risk factors in africa: A systematic review and meta-analysis. Arch Clin Obstet Gynecol Res. 2022;2:1–12.

[62] McDonald JA, Terry MB, Tehranifar P, Racial, Discrimination G. Early life Factors, and chronic physical health conditions in midlife. Womens Health Issues. 2014;24:e53–9. 10.1016/j.whi.2013.09.006. 24345610 10.1016/j.whi.2013.09.006 PMC3905987

[63] Andersson MA, Harnois CE. Higher exposure, lower vulnerability? The curious case of education, gender discrimination, and women’s health. Soc Sci Med. 2020;246:112780. 10.1016/j.socscimed.2019.112780. 31923835 10.1016/j.socscimed.2019.112780

[64] Vigod SN, Rochon PA. The impact of gender discrimination on a woman’s mental health. eClinicalMedicine. 2020;20. 10.1016/j.eclinm.2020.100311. 10.1016/j.eclinm.2020.100311 PMC7082643 32211599

[65] Jacobs EA, Rathouz PJ, Karavolos K, Everson-Rose SA, Janssen I, Kravitz HM, et al. Perceived discrimination is associated with reduced breast and cervical cancer screening: the study of women’s health across the Nation (SWAN). J Womens Health. 2014;23:138–45. 10.1089/jwh.2013.4328. 10.1089/jwh.2013.4328 PMC3922246 24261647

[66] Struckmann et al. Women’s perceptions of gender-based discrimination and its relevance to women’s health: a qualitative study in Burkina Faso, Ghana and Tanzania. forthcoming. 10.1186/s12939-025-02719-5 PMC12853705 41419924

[67] Pennington A, Orton L, Nayak S, Ring A, Petticrew M, Sowden A, et al. The health impacts of women’s low control in their living environment: A theory-based systematic review of observational studies in societies with profound gender discrimination. Health Place. 2018;51:1–10. 10.1016/j.healthplace.2018.02.001. 29482064 10.1016/j.healthplace.2018.02.001

[68] Grown C, Gupta GR, Pande R. Taking action to improve women’s health through gender equality and women’s empowerment. Lancet. 2005;365:541–3. 10.1016/S0140-6736(05)17872-6. 15705464 10.1016/S0140-6736(05)17872-6

[69] United Nations. UNICEF Gender Action Plan, 2022–2025. 2021.

[70] World Health Organization Regional Office of Europe. Health and gender equality: policy brief. World Health Organization. Regional Office for Europe; 2019.

[71] Souza JP, Day LT, Rezende-Gomes AC, Zhang J, Mori R, Baguiya A, et al. A global analysis of the determinants of maternal health and transitions in maternal mortality. Lancet Glob Health. 2024;12:e306–16. 10.1016/S2214-109X(23)00468-0. 38070536 10.1016/S2214-109X(23)00468-0

[72] Yu BCL, Chio FHN, Mak WWS, Corrigan PW, Chan KKY. Internalization process of stigma of people with mental illness across cultures: A meta-analytic structural equation modeling approach. Clin Psychol Rev. 2021;87:102029. 10.1016/j.cpr.2021.102029. 34058604 10.1016/j.cpr.2021.102029

[73] Plouffe V, Bicaba F, Bicaba A, Druetz T. User fee policies and women’s empowerment: a systematic scoping review. BMC Health Serv Res. 2020;20:982. 10.1186/s12913-020-05835-w. 33109172 10.1186/s12913-020-05835-w PMC7590470

[74] Achard V. Jean-Paul dossou: « payer Avant d’accoucher est La première violence faite aux femmes ». Le Monde Afrique. 2019.

[75] World Bank. Maternal mortality ratio (modeled estimate, per 100,000 live births) | Data. 2023. https://data.worldbank.org/indicator/SH.STA.MMRT. Accessed 29 Jan 2023.

[76] Damian DJ, Njau B, Lisasi E, Msuya SE, Boulle A. Trends in maternal and neonatal mortality in South africa: a systematic review. Syst Rev. 2019;8. 10.1186/S13643-019-0991-Y. 10.1186/s13643-019-0991-y PMC6436230 30917874

[77] Brinbaum Y, Safi M, Simon P. Discrimination in france: between perception and experience. In: Beauchemin C, Hamel C, Simon P, editors. Trajectories and origins: survey on the diversity of the French population. Cham: Springer; 2018. pp. 195–222. 10.1007/978-3-319-76638-6_8.

[78] Tayal D. Gender inequality, reproductive rights and food insecurity in Sub-Saharan Africa – a panel data study. Int J Dev Issues. 2019;18:191–208. 10.1108/IJDI-10-2018-0165/FULL/PDF.

[79] Mehrotra S, Kapoor S. Gender discrimination in Asia - Regional perspectives. Glob Soc Policy. 2009;9:197–205. 10.1177/1468018109106891.

[80] Nations U. Gender Inequality Index. UNDP Human Development Reports. https://hdr.undp.org/data-center/thematic-composite-indices/gender-inequality-index#/indicies/GII. Accessed 20 Jan 2023.

[81] CEPAL. Indicators | Gender Equality Observatory. Gender Equality Observatory for Latin America and the Caribbean. https://oig.cepal.org/en/indicators. Accessed 20 Jan 2023.

[82] Justina Demetriades. GENDER EQUALITY INDICATORS: WHAT, WHY AND HOW? Paris: OECD; 2009.

[83] Demetriades J. Gender Indicators: What, Why and How? OECD - based on BRIDGE’s gender and Indicators; 2007.

[84] Kruk ME, Leslie HH, Verguet S, Mbaruku GM, Adanu RMK, Langer A. Quality of basic maternal care functions in health facilities of five African countries: an analysis of National health system surveys. Lancet Glob Health. 2016;4:e845–55. 10.1016/S2214-109X(16)30180-2. 27670090 10.1016/S2214-109X(16)30180-2

[85] Ahmed S, Li Q, Liu L, Tsui AO. Maternal deaths averted by contraceptive use: an analysis of 172 countries. Lancet Lond Engl. 2012;380:111–25. 10.1016/S0140-6736(12)60478-4. 10.1016/S0140-6736(12)60478-4 22784531

[86] Lynch SM, Brown JS. Stratification and inequality over the life course. Handb Aging Soc Sci. 2011;105–17. 10.1016/B978-0-12-380880-6.00008-3.

