# Supplementary material for: Association between self-reported gender-based discrimination and maternal mortality rates: results of an ecological multi-level analysis across nine countries in Sub-Saharan Africa
Source: BMC Public Health. 2025 Oct 21;25:3550. doi: 10.1186/s12889-025-24861-z (PMC12541951; doi:10.1186/s12889-025-24861-z)
Supplement: Supplementary file 1 — Supplementary material 1. [file 12889_2025_24861_MOESM1_ESM.docx]

# Appendix

| **Country** | **Name and Year  of Survey** | |
| --- | --- | --- |
|  | **DHS** | **Afrobarometer** |
| Benin | DHS-VII 2017-18 | R7 2016/2018 |
| Mali | DHS-VII 2018 | R7 2016/2018 |
| Malawi | DHS-VII 2017 | R7 2016/2018 |
| Nigeria | DHS-VII 2018 | R7 2016/2018 |
| Senegal | DHS-VII 2017 | R7 2016/2018 |
| South Africa | DHS-VII 2016 | R7 2016/2018 |
| Uganda | DHS-VII 2016 | R7 2016/2018 |
| Zambia | DHS-VII 2018 | R7 2016/2018 |
| Zimbabwe | DHS-VII 2015 | R7 2016/2018 |

**Appendix 1: Name and year of survey data source per country**

| **Correlation** | School education | High LPI | Difficulties in obtaining medical treatment | Never pay bribes for  medical treatment | Access to water | HIV Prevalence | Health Expenditure | Adolescents’ Fertility Rate |
| --- | --- | --- | --- | --- | --- | --- | --- | --- |
| GBD | .066 | .118 | .106 | -.018 | -.238 | -.134 | -.159 | -.004 |
| School education |  | -.313 | .391 | .183 | -.067 | .669 | .291 | .012 |
| High LPI |  |  | .143 | .045 | .072 | -.140 | -.129 | -.165 |
| Difficulties in obtaining medical  treatment |  |  |  | .724 | -.168 | .433 | -.082 | .117 |
| Never pay bribes for  medical treatment |  |  |  |  | -.223 | .540 | -.025 | .180 |
| Access to water |  |  |  |  |  | -.057 | .373 | -.449 |
| HIV Prevalence |  |  |  |  |  |  | .633 | -.031 |
| Health Expenditure |  |  |  |  |  |  |  | -.422 |

**Appendix 2: Correlations between independent variables – Check for Multicollinearity**

| **Country** | **Region** | **MMR per 100,000 live births** | **GBD [%]** |
| --- | --- | --- | --- |
| BENIN | ALIBORI | 720 | 14,80 |
| BENIN | ATACORA | 299 | 24,70 |
| BENIN | ATLANTIC | 325 | 15,00 |
| BENIN | BORGOU | 403 | 9,30 |
| BENIN | COLLINES | 357 | 9,70 |
| BENIN | COUFFO | 314 | 6,80 |
| BENIN | DONGA | 79 | 0,00 |
| BENIN | LITTORAL | 402 | 7,80 |
| BENIN | MONO | 150 | 10,90 |
| BENIN | OUÉMÉ | 380 | 8,00 |
| BENIN | PLATEAU | 462 | 10,10 |
| BENIN | ZOU | 355 | 12,70 |
| BENIN | Total | 377 | 11,10 |
| MALAWI | NORTHERN REGION | 194 | 7,50 |
| MALAWI | CENTRAL REGION | 340 | 16,00 |
| MALAWI | SOUTHERN REGION | 472 | 9,90 |
| MALAWI | Total | 370 | 12,20 |
| MALI | KAYES | 262 | 3,50 |
| MALI | KOULIKORO | 215 | 2,90 |
| MALI | SIKASSO | 251 | 3,60 |
| MALI | SEGOU | 437 | 3,50 |
| MALI | MOPTI | 293 | 1,50 |
| MALI | TOUMBOUCTOU | 1273 | 3,60 |
| MALI | GAO | 317 | 0,00 |
| MALI | KIDAL | 1992 | 0,00 |
| MALI | BAMAKO | 334 | 2,30 |
| MALI | Total | 407 | 2,80 |
| NIGERIA | NORTH CENTRAL | 247 | 16,00 |
| NIGERIA | NORTH EAST | 1002 | 14,00 |
| NIGERIA | NORTH WEST | 472 | 2,00 |
| NIGERIA | SOUTH EAST | 264 | 18,00 |
| NIGERIA | SOUTH SOUTH | 514 | 14,00 |
| NIGERIA | SOUTH WEST | 258 | 5,00 |
| NIGERIA | Total | 513 | 10,10 |
| SENEGAL | DAKAR | 452 | 6,60 |
| SENEGAL | ZIGUINCHOR | 181 | 0,00 |
| SENEGAL | DIOURBEL | 64 | 5,70 |
| SENEGAL | SAINT-LOUIS | 195 | 8,00 |
| SENEGAL | TAMBACOUNDA | 209 | 23,00 |
| SENEGAL | KAOLACK | 184 | 0,00 |
| SENEGAL | THIÉS | 225 | 7,00 |
| SENEGAL | LOUGA | 156 | 6,00 |
| SENEGAL | FATICK | 350 | 0,00 |
| SENEGAL | KOLDA | 358 | 3,10 |

**Appendix 3: Values of the two main variables (MMR and GBD) according to regions among countries – Part I**

| **Country** | **Region** | **MMR per 100,000 live births** | **GBD [%]** |
| --- | --- | --- | --- |
| SENEGAL | MATAM | 209 | 0,00 |
| SENEGAL | KAFFRINE | 117 | 16,90 |
| SENEGAL | KEDOUGOU | 295 | 17,90 |
| SENEGAL | SEDHIOU | 124 | 0,00 |
| SENEGAL | Total | 214 | 6,30 |
| SOUTH AFRICA | WESTERN CAPE | 0 | 8,20 |
| SOUTH AFRICA | EASTERN CAPE | 467 | 5,20 |
| SOUTH AFRICA | NORTHERN CAPE | 2079 | 2,90 |
| SOUTH AFRICA | FREE STATE | 963 | 3,00 |
| SOUTH AFRICA | KWAZULU-NATAL | 409 | 4,20 |
| SOUTH AFRICA | NORTH WEST | 399 | 1,70 |
| SOUTH AFRICA | GAUTENG | 370 | 6,70 |
| SOUTH AFRICA | MPUMALANGA | 314 | 0,00 |
| SOUTH AFRICA | LIMPOPO | 588 | 14,00 |
| SOUTH AFRICA | Total | 532 | 5,90 |
| UGANDA | KAMPALA | 529 | 4,40 |
| UGANDA | CENTRAL | 466 | 3,10 |
| UGANDA | WEST | 252 | 4,70 |
| UGANDA | NORTH | 335 | 16,10 |
| UGANDA | EAST | 260 | 6,10 |
| UGANDA | Total | 320 | 6,90 |
| ZAMBIA | CENTRAL | 173 | 5,20 |
| ZAMBIA | COPPERBELT | 200 | 1,80 |
| ZAMBIA | EASTERN | 85 | 12,30 |
| ZAMBIA | LUAPULA | 329 | 8,10 |
| ZAMBIA | LUSAKA | 505 | 5,60 |
| ZAMBIA | MUCHINGA | 304 | 9,50 |
| ZAMBIA | NORTHERN | 229 | 18,20 |
| ZAMBIA | NORTH WESTERN | 160 | 2,40 |
| ZAMBIA | SOUTHERN | 67 | 1,80 |
| ZAMBIA | WESTERN | 141 | 10,10 |
| ZAMBIA | Total | 220 | 6,80 |
| ZIMBABWE | MANICALAND | 637 | 5,00 |
| ZIMBABWE | MASHONALAND CENTRAL | 575 | 3,90 |
| ZIMBABWE | MASHONALAND EAST | 510 | 3,40 |
| ZIMBABWE | MASHONALAND WEST | 1065 | 4,80 |
| ZIMBABWE | MATABELALAND NORTH | 1036 | 8,90 |
| ZIMBABWE | MATABELALAND SOUTH | 572 | 7,40 |
| ZIMBABWE | MIDLANDS | 738 | 0,00 |
| ZIMBABWE | MASVINGO | 312 | 2,60 |
| ZIMBABWE | HARARE | 335 | 4,80 |
| ZIMBABWE | BULAWAYO | 834 | 10,50 |
| ZIMBABWE | Total | 659 | 4,50 |

**Appendix 4: Values of the two main variables (MMR and GBD) according to regions among countries – Part II**
